# Using a co-design process to develop an integrated model of care for delivering self-management intervention to multi-morbid COPD people in rural Nepal

**DOI:** 10.1186/s12961-020-00664-z

**Published:** 2021-02-10

**Authors:** Uday Narayan Yadav, Jane Lloyd, Kedar Prasad Baral, Narendra Bhatta, Suresh Mehta, Mark Fort Harris

**Affiliations:** 1 Centre for Primary Health Care and Equity, UNSW, Sydney, Australia; 2 School of Population Health, UNSW, Sydney, Australia; 3 Centre for Research, Policy and Implementation, Biratnagar, Nepal; 4 School of Public Health, Patan Academy of Health Sciences, Kathmandu, Nepal; 5 Department of Pulmonary, Critical Care and Sleep Medicine, B.P. Koirala Institute of Health Sciences (BPKIHS), Dharan, Nepal; 6 Ministry of Health and Population, Kathmandu, Nepal

**Keywords:** A model of care, Co-design, COPD, Health services, Prototype

## Abstract

**Background:**

People with chronic obstructive pulmonary disease (COPD) in Nepal are not receiving adequate support to self-manage their chronic conditions, and primary health care can play a key role in the effective management of these. In this study, we aimed to develop a model of care, using a co-design approach, for delivering evidence-based biomedical and psycho-social care to support self-management for people with multi-morbid COPD in rural Nepal.

**Methods:**

A co-design approach, guided by the five stages of the design thinking model, was used for this study. Layering on “empathize” and “define” phases, we ideated a model of care that was further refined in a “prototype” stage, which included a series of consultative meetings and a 1-day co-design workshop with stakeholders. This co-design process involved a wide range of stakeholders from Nepal, including people with COPD and their families, community representatives, local government representatives, primary care practitioners, community health workers, policymakers, state-level government representatives and academics.

**Results:**

Through our co-design approach, a model of integrated care for delivering evidence-based biomedical and psycho-social care to support self-management for people with multi-morbid COPD was designed. The integrated model of care included: screening of the community members aged > 40 years or exhibiting symptoms for COPD and management of symptomatic patients within primary health care, establishing referral pathways for severe cases to and from secondary/tertiary-level health care and establishing a community-based support system. It involved specific roles for community health workers, patients and their caregivers and community representatives. It was built on existing services and programmes linking primary health care centres and tertiary-level health facilities.

**Conclusion:**

The co-design approach is different from the currently dominant approach of rolling out models of care, which were designed elsewhere with minimal community engagement. In our study, the co-design approach was found to be effective in engaging various stakeholders and in developing a model of care for rural Nepal. This grassroots approach is more likely to be acceptable, effective and sustainable in rural Nepal. Further research is required to test the effectiveness of an integrated model of care in delivering self-management support for people with multi-morbid COPD in rural Nepal.

## Background

Non-communicable diseases ( NCDs) pose a significant challenge to the health care system in many low and middle-income countries (LMICs). The primary health care services in these countries are ill equipped with limited medical supplies and human resources and have significant financial constraints in addressing chronic disease [1, 2]. Nepal, a low-income country in the South Asia, is facing similar challenges in its efforts to address chronic diseases. Chronic obstructive pulmonary disease (COPD) is the most prevalent chronic disease in Nepal, with a prevalence of 11.7% [3].

In the Nepalese context, the number of COPD cases is expected to be underestimated because providers capable of diagnosing COPD represent a small proportion of the health care workforce [4]. The urban areas of Nepal do have health care systems to address the medical needs of people with COPD. In rural areas, primary health care lacks the necessary logistics and human resources to deal with the condition [5]. It also lacks established referral protocols for secondary or tertiary care services [6]. Most people consult local service providers, including pharmacists, health assistants, community medical assistants and medical doctors, in the first instance, who do not have the required infrastructure and training to diagnose and manage the COPD cases. This subsequently delays patients presenting to health facilities served by physicians or specialists. People with COPD often need different types of biomedical and psycho-social care from health professionals, including specialists, community health workers and nurses who have proper training in diagnosing and providing care for COPD patients. The COPD services provided by various levels of health care providers (HCPs) are not properly aligned in Nepal. Among many reasons for this, lack of a model to guide care for NCDs including COPD is a key one. Greater participation of community members in the design and implementation of the model is also needed to make this successful.

Recent political reforms in Nepal have decentralized power to provincial- and local-level governments to address the health and development demands of the population [7]. In recent years, three tiers of governments (federal, state and local level) have attempted to strengthen public health systems (both the peripheral health system and secondary/tertiary hospitals). However, this has not adequately addressed the needs of people with NCDs, including COPD. The increasing burden of COPD and other NCDs posed a major threat to the fragile health system of Nepal. In response, the Government of Nepal (GoN) has led the development of the National Health Policy-2019 [8] and the Nepal Health Sector Strategy Implementation Plan (2016–2021) [9]. These policies have outlined the need for people-centred proactive low-cost interventional approaches that respond to the needs of the communities.

A model of care is defined as a schematic representation of interrelated concepts, assumptions, theories and propositions [10]. Health care systems must transform from fragmented, uncoordinated, single disease-centred services to an integrated care model that can address most clinical, social, psychological and cognitive needs of the people in a more coordinated way if we are to better manage the NCDs [11–13]. In response to these issues, Wagner et al. [14] developed the chronic care model (CCM), which employs six key elements to underpin proactive patient-centred care: self-management support, decision support, redesigning service delivery, clinical information technology, linkages to community resources and health care system organization. There is evidence from systematic reviews that self-management intervention for people with COPD improves quality of life [15, 16] and also reduces the health care utilization without compromising outcomes [17]. There is less evidence of impact on hospitalization and health service costs [17, 18]. Emerging evidence [19, 20] suggests that CCM may not be directly applicable to LMICs. There is a felt need for development of more context-specific models for delivering care for NCDs in LMICs. This vision could be achieved through the active involvement and engagement of users, providers and other stakeholders in co-design of health service delivery and creating supporting environment for the people whom we choose to serve [21–24].

A co-design approach is defined as a “process of collaborative design thinking: a process of joint inquiry and imagination in which diverse people jointly explore and define a problem and jointly develop and evaluate solutions” [25]. Co-design is emerging as the best methodological approach for designing health services [26], and the solutions designed through this process appear more likely to be successful and sustainable [24, 27, 28]. Co-designed service delivery may improve the quality of care and also improve satisfaction with health care services. To date, there has been no study from Nepal that used a co-design approach in designing an integrated model of care for multi-morbid COPD patients. Therefore, we chose to adopt a co-design approach to create change in service delivery for the rural population in Nepal. This co-design process aimed to develop a model of care to deliver comprehensive self-management intervention for multi-morbid COPD people in rural district of Nepal.

## Methods

**Fig. 1:**
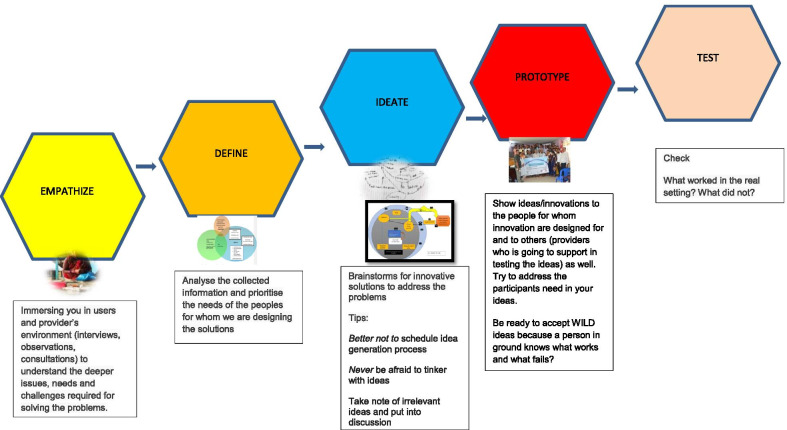
Five stages of design thinking

This study was conducted in two rural municipalities of Sunsari district of Nepal, between August 2018 and August 2020. We used a co-design approach that involved five stages of the design thinking model proposed by the Hasso-Plattner Institute of Design at Stanford [29, 30]. This was chosen because it offered a solution-based approach with clear process modules and has been used effectively [31, 32] in health care improvement. It was readily translated into the Nepalese context. The five stages of the model include: “empathize”, “define”, “ideate”, “prototype” and “test” (Fig. 1). Engagement of the stakeholders (people with COPD and their caregivers, primary health care workers, clinicians, academics, local government officials, state- and central-level policy makers, media persons, etc.) was from the idea inception phase and the stakeholders contributed in all stages of the co-design approach. Stakeholders choose to engage because the research team were able to convince stakeholders about the dearth of evidence on COPD self-management practices at the community level and this motivated them to join this project.

### Empathize

This phase involved a mixed-method study to describe existing self-management practices among the population in rural Nepal and examine how they thought and felt about their health care needs. The quantitative [33, 34] component assessed the level of health literacy, patient activation and self-management practices of rural older people with COPD. The qualitative study [35] explored the facilitators and barriers to self-management among COPD patients from the perspective of both patients and primary HCPs. Immersion and engagement of the principal investigator who was from the same geographical location and could speak local languages enabled an in-depth assessment of the needs of the community. This process helped us to observe the patient's self-management behaviours and the underlying factors which influenced them. We were also able to assess the capacity of the primary health care system and the provider's role and behaviour in delivering care. This stage took 6 months.

### Define

In this stage, we discussed and analysed the quantitative and qualitative data into actionable problem statements. It took 6 months to analyse the quatitative and qualitative data. Our findings revealed problems at the level of patient-family, community and services that needed to be addressed to improve the self-management practices for COPD patients in Nepal. Table 1 shows the key actionable problem statements.

**Table 1:** Key actionable problem statements

Levels of action	Key actionable problem statements
Patient/family level	Poor health literacy of patients/families Inadequate family support Poor emotional wellbeing of people with COPD Limited confidence of patients in communication with health care providers Poor self-management practices Poor level of activation among the people living with COPD
Community level	Complementary and alternative treatment, driven by social network (mostly by Shaman and community members) Poor health literacy at the community level Self-medication practices Cultural practices impeding self-management
Service level	Unavailability of services for COPD at peripheral health system Inadequate capacity of health care providers/community health workers for delivering COPD care Limited skills and expertise of the health care providers in behavioural change Unavailibility of treatment and management guidelines for COPD at the peripheral level Lack of information, educational, communication (IEC) materials for COPD

### Ideate

**Fig. 2:**
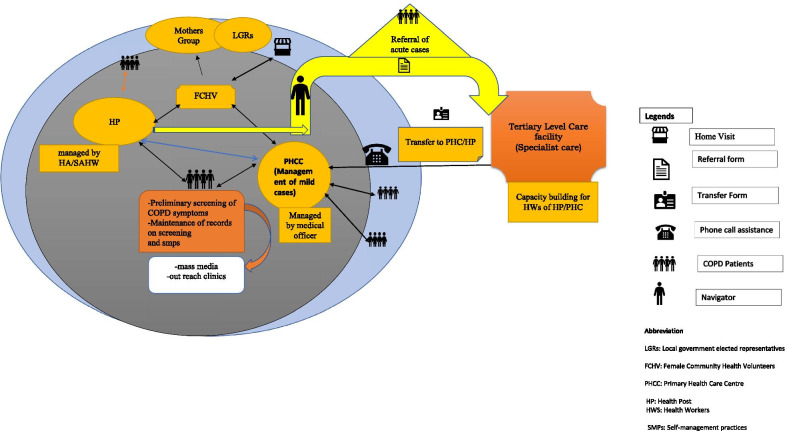
Model of care designed in the ideate stage. *LGRs* local government-elected representatives, *FCHV* Female Community Health Volunteers, *PHCC* Primary Health Care Centre, *HP* health post, *HWS* health workers, *SMPs* self-management practices

Within the context of the problem statements, the team generated “radical design” alternatives [33]. In this process, we used divergent thinking (brainstorming and mind-mapping exercises) followed by convergent thinking in order to synthesize (i.e., refine and integrate) collections of ideas into a cohesive applicable concept. The generated concept was shared with academics (*n* = 4), people with COPD (*n* = 4) and local-/state-level government officials (*n* = 4) of Nepal. These participants agreed to the model of care that we ideated. This entire process led to the development of a conceptual model of care (Fig. 2). We spent 3 months on this stage.

### Prototype

**Fig. 3:**
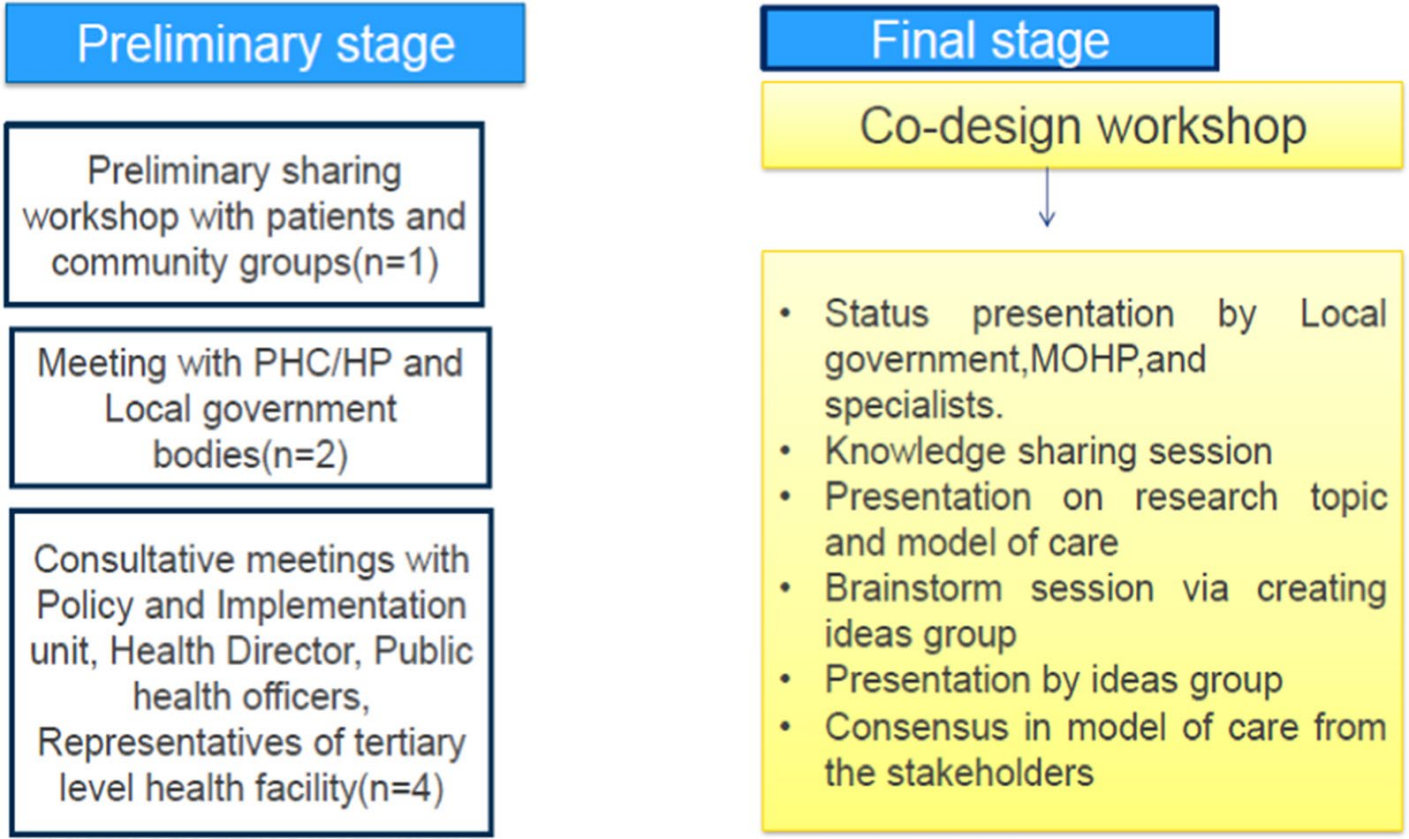
Details on preliminary consultative meetings and a final co-design workshop used in the prototyping stage

In this stage, the model of care was validated for its conceptualization and appropriateness and subsequently refined [33, 36]. The objective of this stage was to initiate evaluation, reflection, and learning and typically to develop a single mature final prototype required for testing or implementation phase. This prototyping included preliminary consultative meetings and a final co-design workshop that provided a “neutral space” to discuss the appropriateness and use of the proposed integrated model of care to address the self-management needs of multi-morbid COPD patients in rural Nepal (Fig. 3). This stage took 4 months.

#### Preliminary consultative meeting

The preliminary consultative meetings engaged four to six participants and ran for 45 min. At these meetings, research findings and a prototype of the model of care were shared with participants. Opportunities were provided for the participants to comment on the shared prototype. The participants from the consultative meeting suggested that only severe cases of COPD be referred to secondary/tertiary hospital, and community health workers will be trained in tertiary-level settings rather than taking specialists to the community. This preliminary meeting helped us to connect participants that helped the research team to design the programme schedule of the final co-design workshop.

#### Co-design workshop

Sixty-eight stakeholders attended a 1-day co-design workshop. Several stakeholder groups were represented including people with COPD and their family members; HCPs including respiratory physicians; local community leaders; representatives from local, provincial and central government; academicians and representatives from local and international Non-Government Organizations (NGOs). This workshop contained a knowledge sharing session, presentations and a brainstorming exercise on the model of care.

**Fig. 4:**
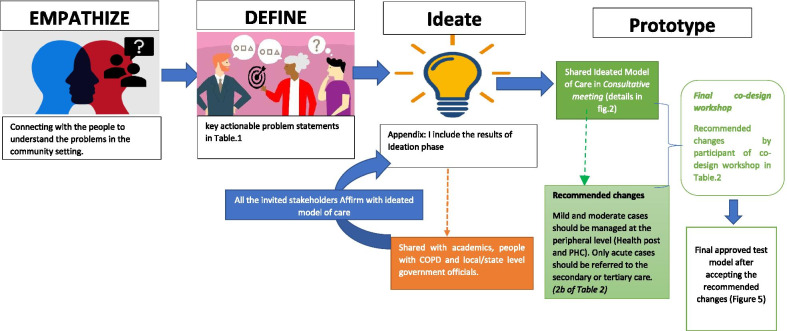
Steps in the co-design process for development of an integrated model of care

In this workshop, we used graphic images in handouts of the prototype (model of care) and a story chart describing a patient journey. These practical tools assisted the participants to enquire into and refine the solutions designed to address the problems encountered by the community. Workshop participants were provided with handouts of the presentation and with the description in the Nepali language to reinforce information given in the presentations. The workshop was largely conducted in Nepali. Two interpreters with a health background (who were fluent in the Nepali, local Maithili and Tharu language) attended to address the language barriers, particularly for the patients and their family members who did not sufficiently understand Nepali. In the brainstorm session, *Idea* groups were created (each group consisting of six participants) and were asked to discuss the issues for 50 min freely. Each ideas group had mixed participants (patients, family members, local government representatives, health care workers, community representatives, policymaker/academicians). During the brainstorm sessions, participants discussed and wrote notes to improve or refine the provided prototype (a model of care). Following this, a representative from each idea group presented their recommendations, changes and suggestions. In this process, service users (patients and their family members) provided insights into what could allow them to respond to services more effectively and identified potential unintended future consequences. The service providers (local government agencies) gave their unique insights into what might work and what would not work for service users (knowledge-based evidence). The entire workshop was facilitated by a health coordinator from the local government body and by the principal investigator of this project. The recommended changes from a co-design workshop are presented in Table 2 and Fig. 4.

**Table 2:** Recommended changes in the proposed integrated model of care in prototype stage

Delivery stages of model of care	Prototype model	Recommended changes	Rationale
1. Screening of population	–	–	–
2. Supporting peripheral health care providers in self-management of the condition	(a) Community health workers will be trained for diagnosis and treatment in the community via hiring specialists	(a) Training to community health workers in tertiary-level settings rather than taking specialists to the community	(a) Rapport building between peripheral-level health care providers and specialists so that they can reach out to specialists very easily in need of any technical support required to manage the cases
	(b) All screened COPD cases will be referred to a tertiary-level health facility	(b) Mild and moderate cases should be managed at the peripheral level (health post and PHC) Only acute cases should be referred to the secondary or tertiary care	(b) There will be no burden of cases at secondary- and tertiary-level care
3. Establishing a referral pathway	(a) No Navigator concept was introduced (b) No involvement in secondary care (district hospital)	(a) Patients expressed the need for a patient navigator who could assist them in navigating the services at both primary and secondary/tertiary care (b) A referral can be done to the secondary- and tertiary-level hospital (district hospital). However, a facility for the respiratory disease at a district hospital was a limitation. Severe cases should be referred directly to tertiary level	(a) Navigators can help to book an appointment and provide accompany to needy ones in a doctor's visit (b) Strengthening of district-level hospitals will benefit the patients in terms of travel cost and time while it would also distribute loads of patients both at secondary and tertiary hospitals This will avoid the delay of receiving emergency health services by patients
4. Community-based care	(a) No involvement of Local elected representatives and other stakeholders like the department of education, agriculture, transport, industry, media, and youth clubs	(a) Involvement of locally elected representatives and other stakeholders like the department of education, agriculture, youth clubs, who could play a valuable role in making health promotion and prevention activities like awareness on COPD, health education at school, street drama and community forums to tackle social issues at the community level. Furthermore, the department of education, agriculture, transport, and industry will develop Strategy to reduce pollutants from industry, transport, and agriculture Legislation to allow local authorities to improve air quality Strategy to create a supportive environment for physical exercise and meditations	(a) Awareness of COPD and tackling social issues as local leaders have social and influential power to promote change. Meaningful engagement of different stakeholders and their interests can speed up the decision-making process, which will help to pursue purpose Perhaps, most importantly, the local government will prioritize and address the issues of people as a whole

### Validation of the co-design process and an integrated model of care

An advisory group was formed, which consists of four potential groups: patients and their care givers (*n* = 4), academics (*n* = 2), clinicians and health care workers (*n* = 4) and policy makers (*n* = 2). Advisory group members participated and engaged in the entire co-design process; they monitored our field activities and have validated the findings and interpretation of the high-valued model of care (Fig. 4) designed for delivering self-management interventions for multi-morbid COPD patients in rural Nepal.

## Results

### Description of the final integrated model of care

**Fig. 5:**
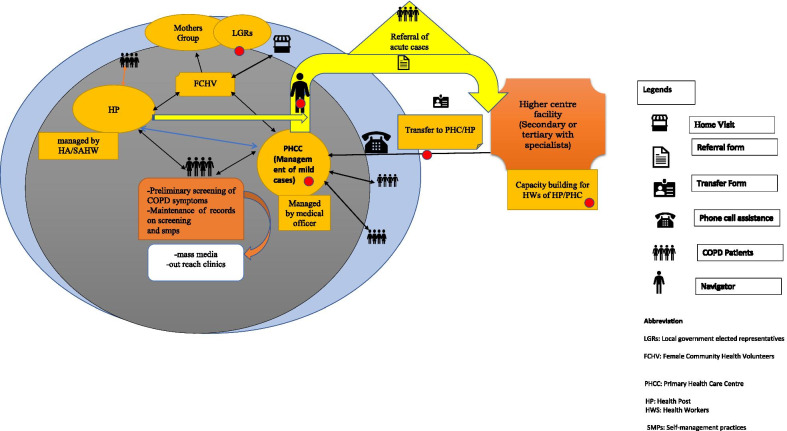
Final integrated model of care after the porotype phase. Red dot indicates the changes made by stakeholders in an integrated model of care during a final co-design workshop. *LGRs* local government elected representatives, *FCHV* Female Community Health Volunteers, *PHCC* Primary Health Care Centre, *HP* health post, *HWS* health workers, *SMPs* self-management practices

A detailed description of the final integrated model of care (Fig. 5) designed to support self-management is described below:

1. Screening of the population
  Questionnaire-based screening of populations aged > 40 years or exhibiting symptoms of COPD will be conducted by community health workers (CHWs) in the community setting of Nepal. People with respiratory disease symptoms will be referred to the primary health care (PHC) facility for the further assessment using peak expiratory flowmeters [37]. The research team will provide support in developing screening tools, building the capacity of primary health care centre (PHCC) doctors in the basic clinical assessment of COPD and managing the records at the peripheral health system.
2. Supporting peripheral health care providers in self-management of COPD
  The HCPs such as medical officer and Auxially Health Workers (AHW)/Health Assistants (HA) of the PHCC/health post (HP) will be trained in: (i) basic clinical assessment of COPD and interpreting the results of peak expiratory flow meters; (ii) managing the mild and moderate COPD cases and referring severe cases to a higher centre's health facility (secondary- or tertiary-level health facility); (iii) motivational interviewing techniques required to engage patients in behaviour change; (iv) providing more patient-centred care that is responsive to patient’s needs. The clinical training of HCPs will be conducted at higher health facilities with the help of trained specialists in the field of respiratory disease. The qualified trainers who have received training of trainers (TOT) will build the capacity of HCPs in motivational interviewing and the patient-centred method [38]. The mid-level health workers (senior AHW/HA) will manage the mild cases under the supervision of the PHCC medical officer, as the medical officer will have more expertise in clinical case management. Where the senior AHW/HA feels difficulty in managing the mild cases, they will refer them to the PHCC, and a medical officer in PHCC will manage them. The treatment at the HP/PHCC level will follow the Nepal Package of Essestional Non-Communicable Diseases (PEN) protocol 3.2 [39] developed by the Government of Nepal. The severe cases of COPD (assessed using PEN protocol) and the patients with multi-morbidity conditions will be referred directly by PHCC/HP to the higher centre health facility (district/zonal or regional referral health facility). HCPs at peripheral health facilities will receive in-phone support from a specialist if needed.
3. Establishing a referral and navigation process
  The health professionals at PHCC/HP will complete a referral form for severe cases, and patients will be directly referred to the nearest higher level facility. The respiratory specialist will provide clinical care to severe COPD cases at the higher level health facility and will link patients with chronic morbidities to the specialist of a particular field if that comes in the picture during the medical examination. In addition to clinical management, clinicians will also deliver health literacy and will empower the patients to manage their conditions.
  Once the clinical management is completed at a higher centre health facility, the patients will be sent back to the PHCC/HP with a transfer form. The transfer form will include the diagnosis report, medical regimen, follow-up and information on support available to patients in their community.
  The cost of the treatment at the higher centre health facility will be covered by the public health insurance provided by the Government of Nepal. The tracking of severe COPD cases between the local health facilities and higher centre health facility will be facilitated by patient navigators. Patient navigators (existing community health workers in government health system trained as navigators) will provide support to severe COPD cases in scheduling appointments with specialists at the higher health care centre, in managing transportation reimbursement for the needy, in navigating patients to the social programmes provided by the local government and in navigating community based primary health care resources. Moreover, the patient navigator will maintain a registration log of patients navigated by them.
4. Community-based support care
  In the community, the COPD patients will be supported by HCPs and female community health volunteers (FCHV). The patients will receive health information on the disease and its risk factors, the benefits of healthy lifestyle behaviours (support quitting the use of tobacco products and engage in physical activity and meditation) from the HCPs, FCHV, mothers group and locally elected representatives. Additionally, FCHV will provide family-based education aiming to address social taboos, create a healthy family environment by restructuring the kitchen environment to reduce indoor household pollution, improve health literacy (at the patient and family level) and help families and caregivers to understand the importance and benefits of their role in patients' health outcomes. FCHV will record the overall health and medication adherence of the patients in the community setting. FCHV will also be responsible for referring the patients to HP/PHCC if they feel patients need support from a health facility.

## Discussion

### Co-design process

To our knowledge, this is the first study outlining the details on the co-design approach used in developing an integrated model of care for delivering comprehensive self-management intervention in a rural area of one district of Nepal. The goal of this co-design approach was to actively involve and engage patients, families, HCPs, academics and other stakeholders in designing a model of care for multi-morbid COPD patients. In this study, through the engagement of a broad range of stakeholders with different expertises and skills, we were able to achieve this goal. We found using a co-design approach was successful in bringing the end-users, policymakers, implementors and researchers together to design a model of care. We found that stakeholders responded concientiously and flexibly to the complex problem statements. This enabled us to develop a user-friendly prototype that was adapted to the context.

Since the inception of this project, various meetings and workshops have been conducted to activate all stakeholders and involve them actively in the design of the model of care. This strategy provided an opportunity for patients to share their experiences and problems and for experts to share their implementation knowledge (what worked, what did not and any unintended consequences according to their past experience) and expertise. Moreover, the adopted approach was unique as it was focused on knowledge sharing and developing the capacity of the recipients to engage in the process, while the traditional methods have used more passive involvement [40]. Our work addressed the recommendations in a recently conducted rapid review [41], which highlighted the need for: building the capacity of end-users, regular communication between the research team and end-users and setting roles for all parties involved in co-design.

This study also confirms the importance of deeper involvement and engagement of HCPs and local government to help them to experience the value of the co-design process and provide a model for them to use in the future work. This strategy is noted in the published literature [23, 42]. Previous studies have shown co-design approaches to be effective in empowering stakeholders [43] and bringing about evidence-based changes at the ground level [44]. Genuine and active engagement of service users and providers in determining the best solutions to fit their needs is a promising approach in contrast to imposing pre-determined ideas to improve health care services. We also anticipate fewer challenges in implementing the model of care as many key stakeholders have been fully engaged and invested in it.

One of the strategies that helped this co-design process to be successfully implemented was meeting with local key stakeholders (people with COPD, family members and caregivers, primary health care workers) in their own local environment. This helped participants to feel a sense of safety in engaging with the research team. We also held face-to-face meetings with the top-level health professionals (federal-, state- and regional-level stakeholders) to secure their engagement. A top-down and bottom-up approach helped us to engage community-level stakeholders and key top-level professionals, including the local government, from the very beginning in the co-design process. There is increasing recognition that such an approach is important in developing the capacity of health facilities, allocating resources and ensuring ownership of the designed solutions [43, 45].

A series of pre-workshops and pre-consultative meetings were conducted with stakeholders prior to the final co-design workshop (prototyping stage). These provided real insights for the stakeholders in the utility of the study and co-design process in addressing identified local problems. Additionally, it created a high level of trust between the stakeholders, including the local government and the research team. A growing number of studies [46–48] indicates that the ‘communication of research findings to non-academic audiences and creating trust between the stakeholders and research team’ are essential components of a change strategy. Moreover, this strategy helped the researchers to collect a prioritised list of changes and suggestions from the users, which in turn helped in the design of the detailed workshop agenda, facilitation plan and engagement strategy. Particularly in developing countries, this strategy could be useful for any co-design process attempting to refine a prototype while engaging a wide range of stakeholders. The co-design process allowed us to build social capital and to develop an evidence-based solution informed by experience.

### Model of care

The model of care was consistent with the CCM. The Nepalese model was designed to address the multi-faceted complex needs of people with multi-morbid COPD conditions. Implementation will be achieved through collaborative partnerships with different stakeholders, building the capacity of the local government to adjust to the change and supporting professional behaviours and attitudes to facilitate the change [49, 50]. This integrated model of care was co-designed using an approach that addressed the different factors [51] (e.g., team climate and readiness, knowledge and beliefs, collaboration/networks, organizational culture, and supportive leadership) known to interact at the level of patient and family, community and health care services, which may otherwise impede adoption in local settings. It is also supported by the Promoting Action on Research Implementation in Health Services (PARIHS) [52] model, which proposes that implementation is more likely to succeed if patients and HCPs are receptive to scientific evidence, organizational readiness for change and mechanisms in place to facilitate intervention. The resultant integrated model of care will help support a more people-centred approach in PHCC and other clinical practice settings.

One of the most obvious concerns is the sustainability of the developed model. Research in implementation science and health service evaluation consistently identifies issues of sustainability [14] and the context in the early failure or success of interventions [16, 17, 19, 24]. Many of these issues may be minimized by applying the learning from the co-design process in programme development and implementation [53]. Of course, funding and resources are also key factors for sustainability in the long run. These may be partially addressed by integrating the model into the routine operations of existing services and programmes and linking it to other policies and programmes such as the above-mentioned PEN programme. The derived model of care will be tested in the rural community setting to see how well it will deliver and address the self-management behaviours among multi-morbid COPD people in Nepal. While we designed a model of care for delivering self-management support to multi-morbid COPD patients in Nepal, there is also a need to develop complementary clinical guidelines for providers that will meet the needs of patients with multi-morbidity.

Despite the strengths of the co-design process, there were some challenges. First, the co-design process created expectations among service users and providers that this programme would be implemented soon after this workshop and comprehensively address the needs of people. This requires the identification of potential funding and the involvement of potential funders in creating and sustaining this change. Second, the co-design process is time-consuming especially with the lead-in time required to build a relationship with participants and involve them in creative processes and to understand the social structures of the community. We also had to work around the hectic work schedules of the different stakeholders. The involvement of key health service managers and planners from the government and cross-networking between the government bodies and including local government helped to address this. Third, engaging patients from a marginalized community was quite challenging. This challenge was addressed by motivating the community leaders and patients from marginalized communities to become involved through frequent visits to their homes. It was also important for researchers and facilitators of the codesign process to be from the same cultural or geographic background as the participants and to have previous experience working with the local community, government officers, policymakers, clinicians and other stakeholders.

## Conclusions

Our co-design approach engaged various stakeholders in designing and shaping solutions to address their complex problems rather than being recipients of the pre-determined solutions. The model of care designed for delivering self-management interventions will address the needs of both users (people with multi-morbid COPD) and health care providers. Our methodological approach could be used by the health care decision makers of Nepal in designing a more people-centred model of care. The most actionable finding from our research was the value of using both top-down and bottom-up approaches to develop an integrated modle of care. This is an important approach that, if repeated in other regions, should help address some of the implementation challenges and health disparities between urban and rural areas in Nepal.

## Data Availability

The datasets used and/or analysed during the current study are available from the corresponding author on reasonable request.

## Abbreviations

AHW: Auxiallary health workers
CCM: Chronic care model
COPD: Chronic obstructive pulmonary disease
FCHV: Female community health volunteers
HA: Health assistants
HCPs: Health care providers
IEC: Information, educational,communication
LMIC: Low and middle-income countries
NCD: Non-communicable diseases
PARIHS: Promoting action on research implementation in health services
PHC: Primary health care
PEN: Package of essestional non-communicable diseases
TOT: Training of trainers
